# Molecular cloning of Peking duck Toll-like receptor 3 (duTLR3) gene and its responses to reovirus infection

**DOI:** 10.1186/s12985-015-0434-x

**Published:** 2015-12-03

**Authors:** Miaotao Zhang, Kaijie Song, Chuanfeng Li, Zongyan Chen, Chan Ding, Guangqing Liu

**Affiliations:** Shanghai Veterinary Research Institute, Chinese Academy of Agricultural Sciences, Shanghai, 200241 People’s Republic of China; College of Veterinary Medicine, Northwest A&F University, Yangling, Shanxi 712100 China

**Keywords:** Peking ducks, Toll-like receptor 3, Innate immunity, Duck reovirus

## Abstract

**Background:**

Toll-like receptors (TLRs) play an important role in detecting pathogen-associated molecular patterns (PAMPs). Among the TLRs, TLR3 is involved in the recognition of double-stranded RNA. This study was designed to explore the relationship between duTLR3 and duck reovirus (DRV) infection.

**Methods:**

In this study, we cloned and performed a molecular characterization of the complete sequence of Peking duck TLR3 (duTLR3). The expression level of duTLR3 was also determined, along with the relative levels of Mx and IFN-α mRNA after DRV infection.

**Results:**

The duTLR3 gene is 2776-bp long and encodes an 895-amino-acid-long protein. Sequence analysis of the product revealed the complete transcript of Peking duck TLR3, including the 88-bp 5′UTR, the 2688-bp coding sequence (ORF), and the 76-bp 3′UTR and poly(A) tail. DuTLR3 was found to share a high amino acid sequence similarity with TLR3 from Jing ding duck (99.6 %), Muscovy duck (97.1 %) and chicken (86.3 %). Additionally, the tissue distribution of duTLR3 suggested that it was abundantly expressed in various tissues, especially in the trachea, esophagus and pancreatic gland. Duck reovirus (DRV) infection resulted in high mRNA expression levels of duTLR3 in the spleen, liver, lung and brain.

**Conclusion:**

These results suggest that duTLR3 may play an important role in anti-viral defense mechanisms.

**Electronic supplementary material:**

The online version of this article (doi:10.1186/s12985-015-0434-x) contains supplementary material, which is available to authorized users.

## Background

The innate immune system is a major contributor to acute inflammation induced by microbial infection or tissue damage. The initial sensing of infection is mediated by innate pattern recognition receptors (PRRs), which include Toll-like receptors (TLRs), RIG-I-like receptors (RLRs), NOD-like receptors and C-type lectin receptors. TLRs play an important role in detecting pathogen-associated molecular patterns (PAMPs). Among the TLRs, TLR3 is involved in the recognition of double-stranded RNA, which is a molecular pattern produced by many viruses and thus can be considered a viral PAMP [1]. TLR3 is normally located in acidic endosomes wherein its luminal ectodomain (ECD) encounters dsRNA. The interaction of dsRNA with the TLR3-ECD leads to receptor dimerization and recruitment of the adapter molecule TRIF to the cytoplasmic domain of TLR3, which is known as a TIR (Toll/interleukin-1 receptor) domain due to its homology with the signaling domains of the IL-1 receptor and plant resistance proteins [2]. TRIF initiates signaling pathways that activate the downstream transcription factors IRF3, AP-1 and NF-κB, which in turn trigger the expression and secretion of type I interferons, inflammatory cytokines and chemokines [3].

Avian immune systems are different from mammalian immune systems [4]. In particular, the natural immune system of waterfowl is significantly different from those of other fowl or mammals. For example, the PRRs of ducks, including TLRs and RLRs, differ from those of other fowl [5–8]. TLR3 plays an important role in defense against viral invasion by up-regulating the expression of IFN-I [9]. TLR3 mainly recognizes dsRNA molecules that are formed during viral genome replication or transcription and localizes exclusively in intracellular vesicles such as endosomes and the endoplasmic reticulum, in which viruses undergo un-coating during infection [10]. Some researchers have shown that TLR3 first recognizes dsRNA or the agonist poly (I: C), and then rapidly induces the production of type IFN-I [11], which induces the expression of some antiviral cytokines. However, the biological characteristics of duck TLR3 and its role in the process of anti-microbial infection are less well understood.

In mammals, 12 members of the TLR family have been identified [12–15], whereas in birds, 10 members of the TLR family have been identified (TLR1LA, TLR1LB, TLR2A, TLR2B, TLR3, TLR4, TLR5, TLR7, TLR15 and TLR21) [16]. However, few members of the duck TLR family have been cloned and characterized. Recently, the dTLR1, dTLR2, dTLR3, dTLR4, dTLR5, dTLR7, dTLR15 and dTLR21 genes were cloned from different duck tissues [17].

In the present study, we cloned and identified Peking duck TLR3 (duTLR3) and analyzed its tissue distribution by quantitative RT-PCR. To explore the relationship between duTLR3 and viral infection, duck reovirus (DRV), which causes serious disease in ducklings within 30 days [18], was selected as a model virus. After infection, the livers, spleens, lungs and brains of the ducks were collected, and the expression level of duTLR3 together with the relative levels of Mx and IFN-α mRNA were determined.

## Results

The full-length duTLR3 cDNA sequence was obtained from Peking duck by homologous cloning and RACE techniques. Sequence analysis of the product revealed the complete transcript of Peking duck duTLR3, including the 88-bp 5′UTR, the 2688-bp coding sequence (ORF), and the 76-bp 3′UTR and poly(A) tail. The longest open reading frame of duTLR3 began at nucleotide 89 and terminated at nucleotide 2776, encoding an 895-amino-acid-long polypeptide. Sequence alignments were performed to determine the percentage homology of duTLR3 with other known TLR3 genes. DuTLR3 shared a high amino acid sequence similarity with the sequences of Jing ding duck (99.6 %), Muscovy duck (97.1 %) and chicken (86.3 %), and moderate similarity with the sequences of human and mouse TLR3 genes (62.3 and 60.0 %, respectively) (Additional file 1: Figure S1).

**Fig. 1:**
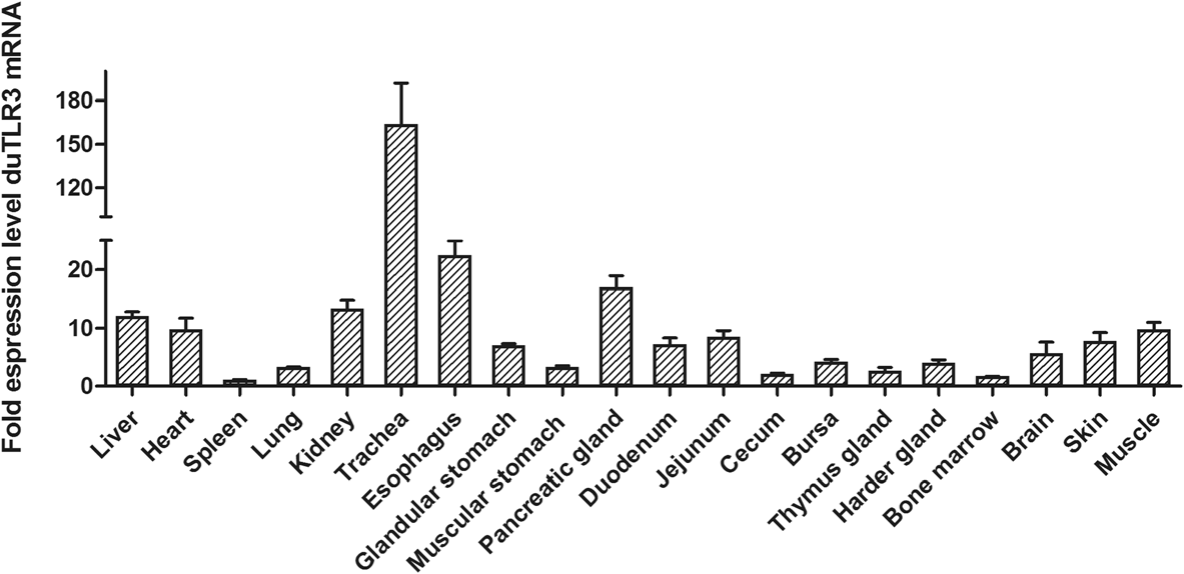
The tissue distribution of duTLR3 transcripts in healthy Peking ducks. We used β-actin as a control gene to quantify the expression levels of duTLR3. Each result represents the level of target gene mRNA relative to that in the spleen, expressed as the mean ± SD of triplicate analyses by quantitative real-time PCR

The duTLR3 expression level in different normal duck tissues was measured by qRT-PCR. As shown in Fig. 1, duTLR3 mRNA was constitutively expressed in almost all of the tissues examined. Among them, the highest expression of duTLR3 was detected in the trachea, esophagus and pancreatic gland; moderate levels were found in the liver, heart and kidney; and lower levels were observed in the spleen, lung, glandular stomach, duodenum, jejunum, cecum, bursa, thymus gland, harder gland, bone marrow, brain, skin and muscle (Fig. 1).

**Fig. 2:**
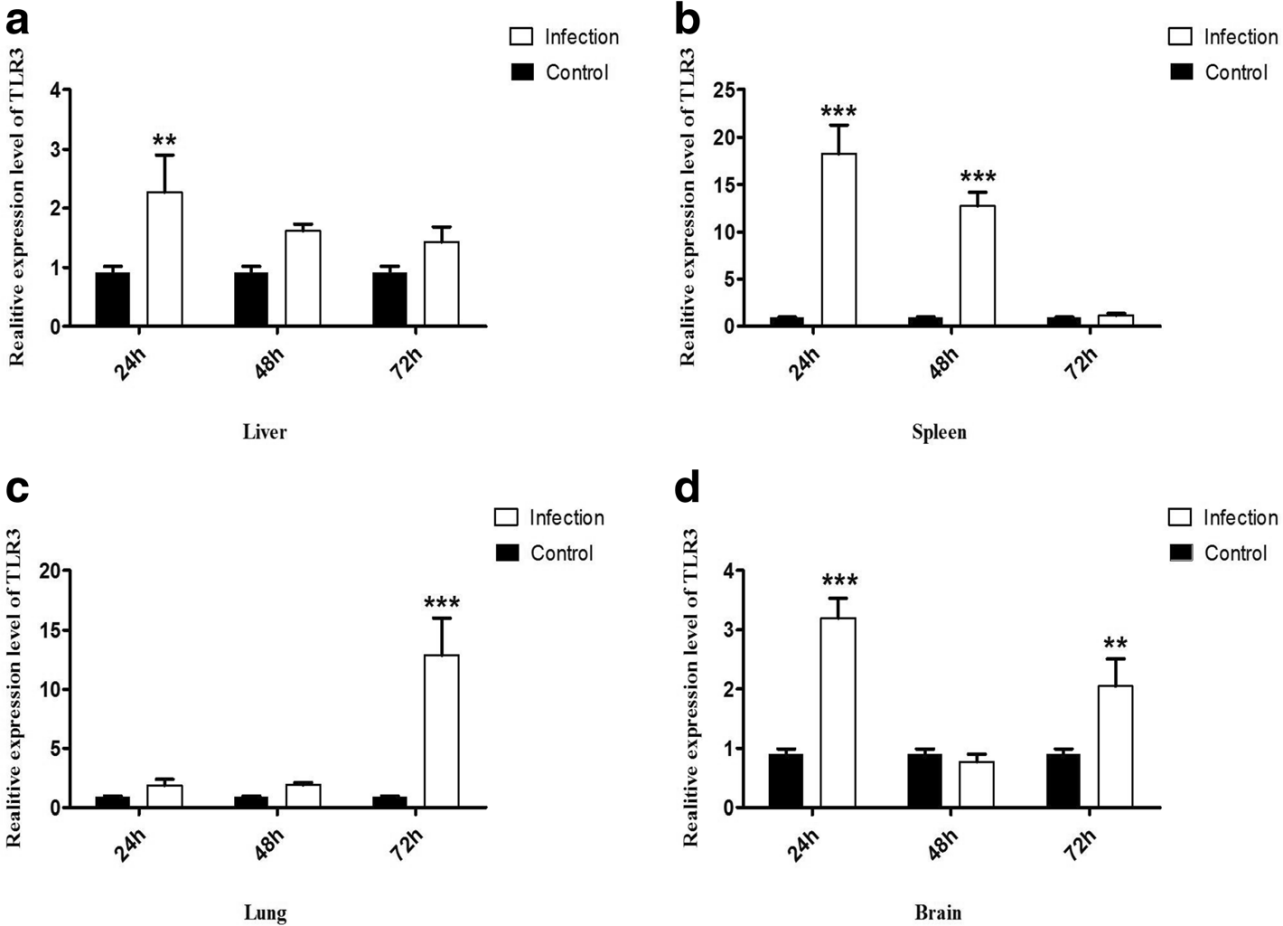
The expression profiles of Peking ducking Toll-like receptor 3 (duTLR3) mRNA in virus-infected tissue by quantitative real-time PCR. **a** Relative Toll-like receptor 3 (TLR3) mRNA expression pattern in liver, (**b**) spleen, (**c**) lung and (**d**) brain. The controls were inoculated with PBS; the experimental ducks were infected with DRV (TH11). Each bar represents the level of target gene mRNA relative to the control group. Asterisks indicate a statistically significant difference (**P < 0.05 and *** *P* < 0.01, student’s *t*-test) between the experimental group and the control group used for normalization. Error bars indicate SD

To further understand the modulation of the expression of duTLR3 in response to challenge with DRV (TH11), qRT-PCR was employed to detect the mRNA levels of duTLR3 in the liver, spleen, lung and brain, from 0 to 72 h after challenge. The mRNA expression in the spleen and liver was up-regulated significantly at 24 h (18.23-fold and 2.27-fold, respectively), 48 h (12.76-fold and 1.61-fold), and 72 h (1.2-fold and 1.43-fold) after challenge relative to the spleen and liver of the controls at all time points (Fig. 2). The duTLR3 transcripts in the lungs peaked at 72 h (12.89-fold) (Fig. 2), but the mRNA expression of duTLR3 in the brain increased 3.20-fold at 24 h, returned to normal levels at 48 h (0.77-fold) (Fig. 2), and increased by 2.05-fold at 72 h after challenge.

**Fig. 3:**
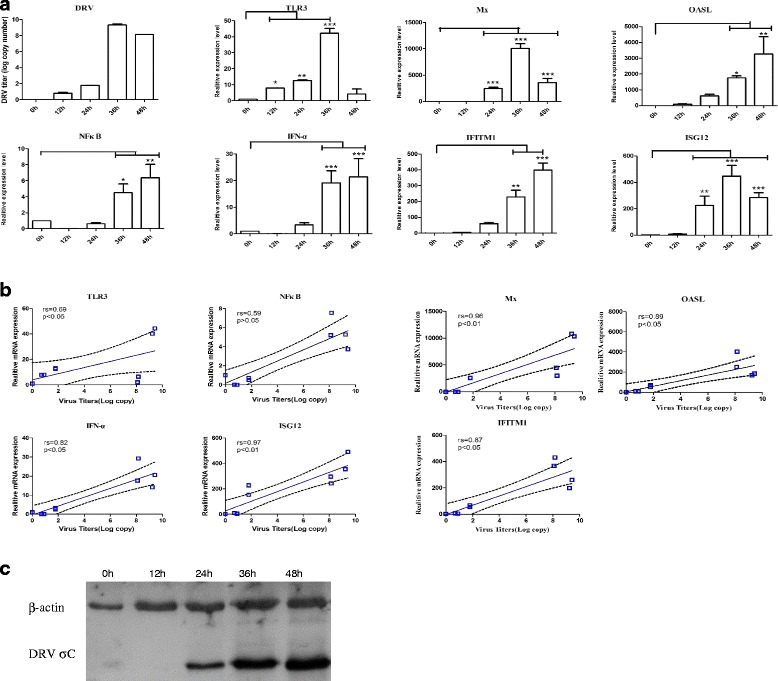
DRV (TH11) infection induces the expression of TLR3, IFN-α, NFκB, Mx, OASL, ISG12 and IFITM1 in CCL-141 cells. **a** Gene responses in duck embryo fibroblast cells incubated with DRV (TH11) at a multiplicity of infection (0.1 MOI) and harvested at the indicated times. The mRNA levels are presented as the mean ± SD. Asterisks indicate significant differences in the virus-infected group compared with the uninfected group (*P < 0.05, ** *P* < 0.01 and *** *P* < 0.001 by one-way ANOVA). **b** Correlation between gene expression and viral replication in CCL-141 cells. Statistical analyses were performed using Spearman’s rank correlation coefficient test. Correlation coefficients (rs) and *P* values are indicated in each graph. **c** DEF cells were infected with DRV (TH11) and harvested at the indicated times. The cell lysates were analyzed by Western blot using the indicated antibodies. The results are representative of three independent experiments

To explore whether duTLR3, transcription factors, interferon-stimulated genes and interferon can respond to DRV infection, the correlation between viral replication and the expression of duTLR3, IFN-α, NFκB, Mx, OASL, ISG12 and IFITM1 in CCL-141 cells (duck embryo fibroblast cells) infected with DRV strain TH11 was assessed. qRT-PCR was used to detect the expression of the genes in CCL-141 cells at 12, 24, 36 and 48 h after DRV infection. The replication level of DRV TH11 was evaluated through detecting the expression level of DRV σC protein by western blot, as expected, the virus replication level is higher and higher with the extension of infection time (Fig. 3c). The results showed that DRV could induce significant changes in duTLR3 expression and modulate the expression of other cytokines, accompanied by the proliferation of DRV in DEF cells (Fig. 3a). In addition, the expression levels of TLR3, IFN-α, NFκB, Mx, OASL, ISG12 and IFITM1 were dependent on the viral titer (Fig. 3b).

**Fig. 4:**
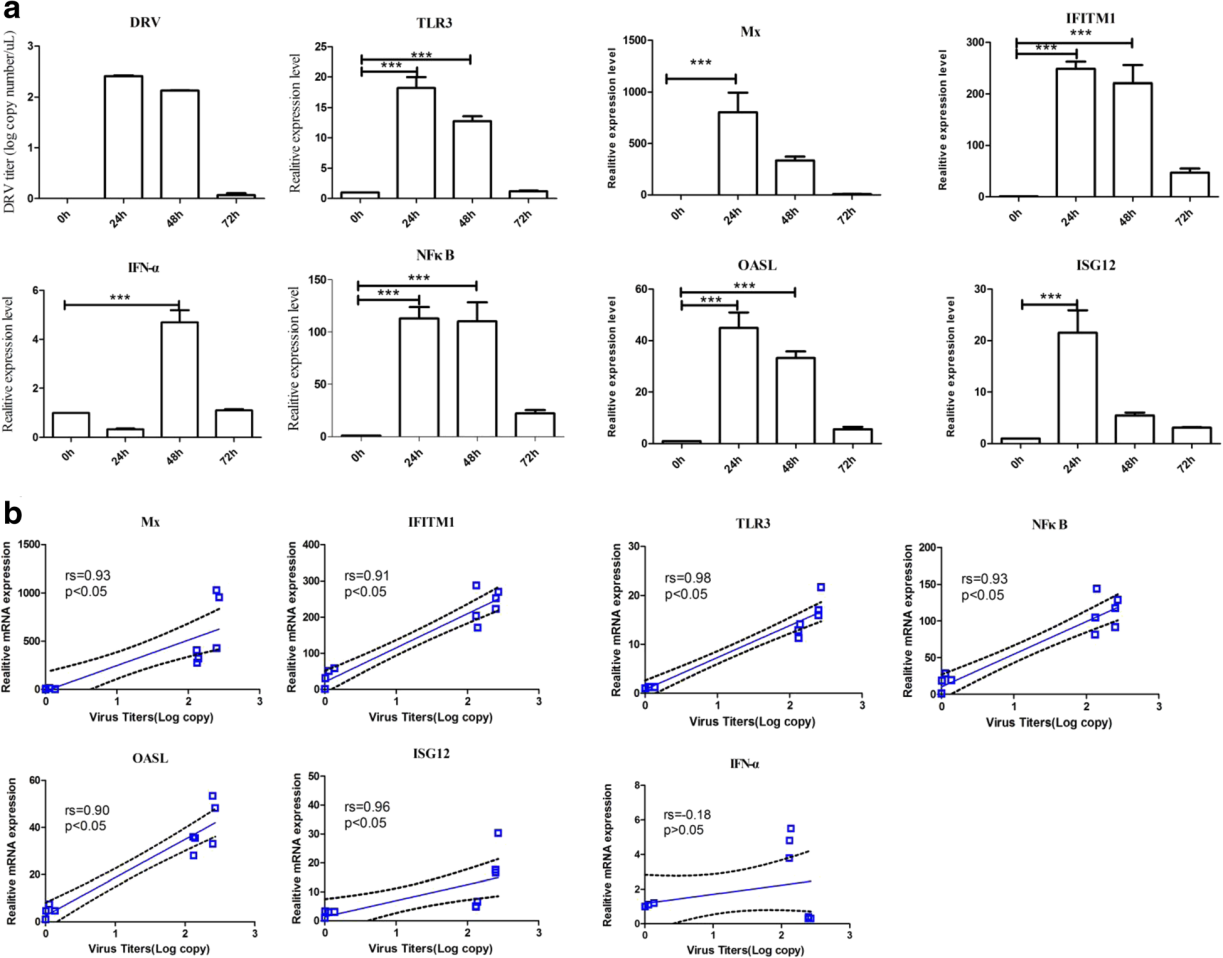
DRV (TH11) infection induces the expression of TLR3, IFN-α, NFκB, Mx, OASL, ISG12 and IFITM1 in the spleens of Peking ducklings. **a** Gene responses in spleens infected with DRV (TH11) at 10^6^ ELD_50_/0.2 mL. The mRNA levels are presented as the mean ± SD. Asterisks indicate significant differences in the virus-infected group compared with the uninfected group (*P < 0.05, ** *P* < 0.01 and *** *P* < 0.001, one-way ANOVA analysis). **b** Correlation between gene expression and viral replication in the spleen. Statistical analyses were performed using Spearman’s rank correlation coefficient test. Correlation coefficients (rs) and *P* values are indicated in each graph. The samples were collected from infected ducklings at 0, 24, 48 and 72 h, respectively

To evaluate organ-specific host responses to DRV infection, we measured the cytokine levels in the liver, lung and brain. The results revealed a weak host cytokine response in the liver, lung and brain due to the lower viral replication; however, this effect was not observed in the spleen. Subsequently, we measured the expression levels of IFN-α, NFκB, Mx, OASL, ISG12 and IFITM1 in Peking duck spleens to evaluate the host innate immune response of ducks infected with DRV (Fig. 4a). In addition, the correlation between viral replication and gene expression in the spleens of ducks infected with DRV was determined. As shown in Fig. 4b, the expression of NFκB, Mx, OASL, ISG12 and IFITM1 correlated positively with viral replication in Peking duck.

## Discussion

Many recent reports have demonstrated that host TLR3 plays an important role in the process of antiviral infection. To understand the role of duck TLR3 during the course of DRV infection in ducks, in the present study, we cloned the complete duTLR3 gene from Peking duck and evaluated its anti-viral properties. The sequencing results showed that the TLR3 gene had a high sequence similarity with other avian sequences, demonstrating similarity values of more than 97 % among members of the duck family and more than 86 % with chicken.

The expression levels of duTLR3 in different normal duck tissues were measured by qRT-PCR. The results showed that duTLR3 mRNA was constructively expressed in almost all of the tissues examined. Among them, the highest duTLR3 expression was detected in the trachea, esophagus and pancreatic gland; moderate levels were found in the liver, heart and kidney; and lower levels were observed in the spleen, lung, glandular stomach, duodenum, jejunum, cecum, bursa, thymus gland, harder gland, bone marrow, brain, skin and muscle. These results are similar to those reported for Muscovy duck tissues but are slightly different from ChTLR3 [19, 20]. We hypothesized that the different expression profiles of TLR3 between duck and chicken might influence their susceptibility to different pathogens.

To further understand the modulation of the expression of duTLR3 following challenge with DRV (TH11), qRT-PCR analysis was employed to measure the mRNA levels of duTLR3 in the liver, spleen, lung and brain. The data revealed that duTLR3 expression was significantly up-regulated in the spleen and liver at 24, 48 and 72 h after infection relative to the spleen and liver of the controls at all time points. The expression of duTLR3 in the lungs peaked at 72 h, but the expression in the brain increased 3.20-fold at 24 h, returned to normal levels at 48 h, and increased by 2.05-fold at 72 h. We cannot explain the different expression pattern of duTLR3 in duck brain. To explore whether duTLR3, transcription factors, interferon-stimulated genes and interferon can respond to DRV infection, the correlation between viral replication and the expression of duTLR3, IFN-α, NFκB, Mx, OASL, ISG12 and IFITM1 in CCL-141 cells infected with DRV strain TH11 was determined. qRT-PCR was used to detect the expression of the genes in CCL-141 cells at 12, 24, 36 and 48 h after DRV infection. The results showed that DRV could induce significant changes in duTLR3 expression, affect the expression of cytokines, and induce the proliferation of DRV in DEF cells. In addition, the expression levels of TLR3, IFN-α, NFκB, Mx, OASL, ISG12 and IFITM1 were viral titer-dependent, indicating that the expression of these cytokines correlated positively with viral replication. It is worth noting that the expression levels of duTLR3, NFκB, Mx, OASL, ISG12 and IFITM1 were up-regulated in CCL-141 cells at 36 h after DRV infection. However, at 48 h post-infection, the expression profiles of these innate immune response genes changed.

To evaluate organ-specific host responses to DRV infection, we measured cytokine levels in the liver, lung and brain. The results revealed a weak host cytokine response in the liver, lung and brain, likely due to the lower viral replication, but different results were obtained for the spleen. In addition, the correlation between viral replication and gene expression in the spleens of ducks infected with DRV was analyzed to assess the host response to DRV. The results showed that the expression levels of NFκB, Mx, OASL, ISG12 and IFITM1 correlated positively with viral replication in Peking duck. Of course, if we could demonstrated this correlation with the western bloting at protein level, our conclussions would be more persuasive. However, because of the lacking of the specific antibodies against duck cytokines, we can’t do these experiments at present. Even so, the results of this paper were positive, and will be helpful for our further studying on the innate immunity of duck.

## Conclusion

In conclusion, the cDNA sequence of Peking duck TLR3 was cloned and sequenced for the first time. The results showed that duTLR3 is widely expressed in the duck and exhibits different tissue-specific expression patterns. The expression levels of duTLR3 and IFN-α, NFκB, Mx, OASL, ISG12 and IFITM1 were up-regulated rapidly in ducks in response to DRV infection.

## Methods

### Reagents

The following antibodies were used in this study: anti-β-actin (CWBIO, China) and rabbit anti-DRV/σC polyclonal antibodies (Shanghai Veterinary Research Institute, Chinese Academy of Agricultural Sciences, China).

### Virus strain

The duck reovirus strain TH11 used in this study was isolated from ducks in Anhui Province, China, in 2011 [21]. The virus titers of the TH11 strain were determined using the EID_50_ method. Allantoic fluid pooled from multiple eggs was clarified by centrifugation and frozen in aliquots at −70 °C.

### Cell line

Duck embryo fibroblast cells (DEFs, CCL-141) were purchased from the American Type Culture Collection (Manassas, VA). The cells were cultured in EMEM supplemented with 10 % fetal bovine serum.

### Virus infection

For the cell infections, CCL-141 cells were washed and infected with DRV at an MOI of 0.1. After incubation for 60 min at 37 °C, the cells were cultured in EMEM supplemented with 2 % fetal bovine serum. For the duck infections, 1-day-old healthy Peking ducks were confirmed serologically negative for DRV by ELISA (developed by Shanghai Veterinary Research Institute, Chinese Academy of Agricultural Sciences, China). After 4 weeks, 5 uninfected ducks were euthanized, and tissues were collected for tissue distribution analyses, including the liver, heart, spleen, lung, kidney, trachea, esophagus, glandular stomach, muscular stomach, pancreatic gland, duodenum, jejunum, cecum, bursa, thymus gland, harder gland, bone marrow, brain, skin and muscle. 4-week-old ducks were divided into 2 groups. In group 1, 15 ducklings were inoculated with DRV TH11. All of the ducklings were challenged in the quadriceps muscle of both legs with a viral suspension containing 10^6^ EID_50_ DRV TH11. In group 2, 15 ducklings were inoculated with 0.2 mL of PBS as the negative control. At 24, 48 and 72 h post-inoculation, 5 individuals from each group were euthanized, and liver, spleen, lung and brain tissues were harvested immediately for RNA extraction. This study was approved by the ethics committee of Shanghai Veterinary Research Institute, the Chinese Academy of Agricultural Sciences (CAAS).

### Western blotting

For the Western blot analysis, CCL-141 cells were harvested at 12, 24, 36 and 48 h post-DRV infection and lysed for protein collection. The proteins were separated in a 10 % SDS-polyacrylamide gel, transferred onto a nitrocellulose membrane, and incubated with the indicated antibodies.

### Cloning of the duTLR3 cDNA sequence

According to the reported sequence of the Anas platyrhynchos TLR3 gene (GenBank accession number: JQ910167.1), primers TLR3-F/ TLR3-R were designed to amplify the CDS of the Peking duck TLR3 gene (Table 1). To synthesize duTLR3 cDNA, mRNA isolated from Peking duck peripheral blood mononuclear cells (PBMCs) was used as the template. The cycling parameters were as follows: 95 °C for 5 min, 35 cycles at 94 °C for 45 s, 55 °C for 30 s and 72 °C for 3 min, and a final extension at 72 °C for 10 min. The polymerase chain reaction (PCR) product was cloned into the pMD19-T-simple vector (TaKaRa, Japan) and sequenced.

**Table 1:** Primers used in this study

Primer	Sequence of oligonucleotide (5′ → 3′)	Application
TLR3-F	AGCCATGGGAAGTGATATTCT	Gene cloning
TLR3-R	CGCTCACCGTGCTTTACTATTAGAT	
3′ RACE outer primer	TACCGTCGTTCCACTAGTGATTT	RACE PCR
TLR3-3GSP1	GGATAGCTTTCTACTGGAACATT	
3′ RACE inner primer	CGCGGATCCTCCACTAGTGATTTCACTATAGG	
TLR3-3GSP2	AACGGGACTTTGAAGCAGGCGTATC	
5′ RACE outer primer	CATGGCTACATGCTGACAGCCTA	
TLR3-5GSP1	GTTGCAATCCTAAATTTGCTGACTT	
5′ RACE inner primer	CGCGGATCCACAGCCTACTGATGATCAGTCGATG	
TLR3-5GSP2	CAGGGGCAAATTTTTGCACAGTTC	
TLR3-5GSP1	GTTGCAATCCTAAATTTGCTGACTT	
qTLR3-F	ATGTCATGCAAACCTGACCA	Real-time quantitative PCR
qTLR3-R	CCAGGGTCTTGAAAGGATCA	
qNFκB-F	TGTCAGCCTTCTGGATCGCCACG	
qMx-F	CATGGTTGTGAAGTGTCGTGGTC	
qMx-R	TATTGTGGGTGTGCCTCGTCTGT	
qNFκB-F	TGTCAGCCTTCTGGATCGCCACG	
qNFκB-R	GCCCGCCAAGGGGATGTTTTCTA	
qIFN-α-F	GGGCCCCGCAACCTT	
qIFN-α-R	CTGTAGGTGTGGTTCTGGAGGAA	
qISG12-F	AAAATGGCTGACCGAAACGT	
qISG12-R	TGTGTGAAGCAAGCGAACCT	
qOASL-F	GACCAACACACACTGCAGCACTA	
qOASL-R	CCGTAGCCGCAGAAGCA	
qIFITM1-F	CACCGCCAAGTACCTGAACA	
qIFITM1-R	CGATCAGGGCGATGATGAG	
qACTß-F	CCCCATTGAACACGGTATTGTC	
qACTß-R	GGCTACATACATGGCTGGGG	

### RACE PCR

Rapid amplification of cDNA ends (RACE) was performed using 3′-full/5′-full RACE kits (TaKaRa, Japan) as previously described. The outer PCR amplification was performed with the primer TLR3-3GSP1 and the 3′ RACE outer primer (Table 1) using 2 μL of cDNA template. The outer PCR product was then used as template for the inner PCR reaction with primer TLR3-3GSP2 and the 3′ RACE inner primer (Table 1). For 5′ RACE, total RNA was first processed using a 5′-full RACE kit through dephosphorylation, 5′ cap structure removal, 5′-RACE adaptor connection and reverse-transcription reaction steps to provide a template for the outer PCR [22, 23]. The outer and inner PCR amplifications for 5′ RACE were performed similarly to those described for the 3′ RACE, using TLR3-5GSP1 and 5′ RACE outer primer for the outer PCR reaction and 5′ RACE inner primer (Table 1) for the inner PCR reaction.

### Bioinformatics analysis

Sequence homology was obtained by BLAST program analysis using the nucleotide database of the National Center for Biotechnology Information website (http: //www.ncbi.nlm.nih.gov/blast). Amino acid sequences were aligned using Clustal Omega (http:// www. ebi.ac. UK / Tools /msa /clustalo) and edited with BOXSHADE (http://www.ch.embnet.org/ software). The Simple Modular Architecture Research Tool (http://smart.Embl-heidelberg.de) was used to predict the domain structure of duTLR3 [24].

### Gene expression of duTLR3 and innate immune response genes in control and DRV-infected ducks

Total RNA was extracted from Peking duck tissues with TRIzol (Invitrogen, USA) according to the manufacturer’s instructions, and total RNA (2 μg) was reverse-transcribed using the First-Strand synthesis of cDNA system (Promega). Quantitative real time PCR (qRT-PCR) was performed using SYBR® Premix Ex Taq™ (Tli RNaseH Plus) (TaKaRa, Japan). The primers are listed in Table 1, and the PCR conditions were as follows: 1 cycle at 95 °C for 5 min, followed by 40 cycles at 95 °C for 15 s and 60° for 30 s. The dissociation curves were generated by increasing the temperature of the samples incrementally from 55 to 100 °C as the final step of the PCR.

### Calculations and statistics, and nucleotide sequence deposition

The relative expression ratios of the target gene in the tested group versus the control group were calculated by the 2^−∆∆Ct^ method using the duck housekeeping gene beta-actin (ACTB; EF667345.1) as the endogenous reference gene to normalize the level of target gene expression. Standard deviations were calculated based on the relative expression ratios of 3 replicates for each gene measured. Statistical analyses of the host cytokine response in Peking duckling spleen infected with DRV (TH11) were performed using GraphPad Prism 5 software (GraphPad Software Inc., San Diego, CA). *P* < 0.05 was considered significant.

The cDNA sequence of the Peking duck duTLR3 gene was deposited in GenBank under accession number KM434239.

## Additional file

Additional file 1: Figure S1.
**Amino acid alignment of Peking duck**, ***Anas platyrhynchos, Cairina moschata, Gallus, Homo sapiens***, **and** ***Mus musculus*** **TLR3.** Alignment was performed using the CLUSTAW program and edited with BOXSHADE. Black boxes indicate amino acid identity; gray boxes indicates similarity (50 % threshold). LRR, leucine rich repeat. TIR, toll-interleukin 1 receptor signaling domain. The TLR3 sequences are shown for Muscovy duck (Cai), human (Hom), mouse (Mus), chicken (Gal), Jinding duck (Ana), and peking duck (Pek). (DOC 51 kb)

## Abbreviations

DEFs: duck embryo fibroblast cells
DRV: duck reovirus
ECD: ectodomain
PAMPs: pathogen-associated molecular patterns
PBMCs: peripheral blood mononuclear cells
PRRs: pattern recognition receptors
RACE: rapid amplification of cDNA ends
RLRs: RIG-I-like receptors
TLRs: Toll-like receptors
