# Supplementary material for: Molecular cloning of Peking duck Toll-like receptor 3 (duTLR3) gene and its responses to reovirus infection
Source: Virol J. 2015 Dec 3;12:207. doi: 10.1186/s12985-015-0434-x (PMC4668636; doi:10.1186/s12985-015-0434-x)
Supplement: Additional file 1: Figure S1. — Amino acid alignment of Peking duck, Anas platyrhynchos, Cairina moschata, Gallus, Homo sapiens, and Mus musculus TLR3. Alignment was performed using the CLUSTAW program and edited with BOXSHADE. Black boxes indicate amino acid identity; gray boxes indicates similarity (50 % threshold). LRR, leucine rich repeat. TIR, toll-interleukin 1 receptor signaling domain. The TLR3 sequences are shown for Muscovy duck (Cai), human (Hom), mouse (Mus), chicken (Gal), Jinding duck (Ana), and peking duck (Pek). (DOC 51 kb) [file 12985_2015_434_MOESM1_ESM.doc]

Pek 1 MGSDILCWKSLSLRLVSVCLLCASVGKQCQIRNTMADCSHLKLTQIPSDLPNNITGLDISHNQLKKLCHANLTRYSKLVYLNAGYNIISKLQPELCKNLP

Ana 1 MGSDILCWKSLSLRLVSVCLLCASVGKQCQIRNTMADCSHLKLTQIPSDLPNNITGLDISHNQLKKLCHANLTRYSKLVYLNAGYNIISKLQPELCKNLP

Cai 1 MGTDILCWNSLSLRLVSVCLLCASVGKQCQIRNTMADCSHLKLTQIPSDLPNNITGLDISHNQLKKLCNANLTKYSKLVYLNAGYNIISKLQPELCKNLP

Gal 1 ------------------------------------------------------MLEEVKLAELKKLDPENLTEYSNLIYLNAGYNIISKLKPGLCKNLP

Hom 1 MR-QTLPCIYFWGGLLPFGMLCASSTTKCTVSHEVADCSHLKLTQVPDDLPTNITVLNLTHNQLRRLPAANFTRYSQLTSLDVGFNTISKLEPELCQKLP

Mus 1 MKGCSSYLMYSFGGLLSLWILLVSSTNQCTVRYNVADCSHLKLTHIPDDLPSNITVLNLTHNQLRRLPPTNFTRYSQLAILDAGFNSISKLEPELCQILP

Pek 101 LLQILVLEHNQLHELPDGAFASCTNLTELNLGYN-IIDVKNDPFKTLENLNILDLTHNHLKSANLGLQQQLKNLRELVLCGNQITELKKEDLSFLSNTSL

Ana 101 LLQILVLEHNQLHELPDGAFASCTNLTELNLGYN-IIDVKNDPFKTLENLNILDLTHNHLKSANLGLQQQLKNLRELVLCGNQITELKKEDLSFLSNTSL

Cai 101 LLQILVLEHNQLHELPDGAFVSCTNLTELNLGYN-IIDIKNDPFKTLENLNILDLTHNHLKSANLGLQQQLKNLRERVLCGNQIAELKKEDLIFLSNTSL

Gal 47 LLQILKLEHNQLHELPDGVFASCSNLTELNLGYN-IIEVKNDPFKTLENLNILDLSHNHLKSANLGLQQQLKNLRELVLYSNQITELNKEDLKFLSNTSL

Hom 100 MLKVLNLQHNELSQLSDKTFAFCTNLTELHLMSNSIQKIKNNPFVKQKNLITLDLSHNGLSSTKLGTQVQLENLQELLLSNNKIQALKSEELDIFANSSL

Mus 101 LLKVLNLQHNELSQISDQTFVFCTNLTELDLMSNSIHKIKSNPFKNQKNLIKLDLSHNGLSSTKLGTGVQLENLQELLLAKNKILALRSEELEFLGNSSL

Pek 200 NSLDLSSNPLKEFHTGCLRAIGNLFGLILNNVELGENRTEKLCTELSNTAIQNLSLSHVKLSYINKTTFQGLQGTNLTVLNLSKNYLSVIEDNSFQWLSN

Ana 200 NSLDLSSNPLKEFHTGCLRAIGNLFGLILNNVELGENRTQKLCTELSNTAIQNLSLSHVKLSYINKTTFQGLQGTNLTVLNLSKNYLSVIEDNSFQWLSN

Cai 200 SSLDLSSNPLKEFHTGCLRTIGNLFGLILNNVELGENRTEKLCTELSNTAIQNLSLSHVKLSYINKTTFQGLQGTNLTVLNLSKNYLYVIEDDSFQWLSN

Gal 146 NSLDLSSNPLKEFHTGCLHAIGNLFGLILNNVELGENRTKKLCTELSDTAIQNLSLSHVKLSHINRLTLQGLQGTNLTVLNLSKNSLSVIEDDSFQWLSK

Hom 200 KKLELSSNQIKEFSPGCFHAIGRLFGLFLNNVQLGPSLTEKLCLELANTSIRNLSLSNSQLSTTSNTTFLGLKWTNLTMLDLSYNNLNVVGNDSFAWLPQ

Mus 201 RKLDLSSNPLKEFSPGCFQTIGKLFALLLNNAQLNPHLTEKLCWELSNTSIQNLSLANNQLLATSESTFSGLKWTNLTQLDLSYNNLHDVGNGSFSYLPS

Pek 300 LEYLNLKDN-SIHVSSHSFYGLSSVKHLNLINSLTG---------KIEDFSFHWLYHLEYLLMDNNNFPRITTNMFTGLNNLKYLSLSNCNINLQRITNT

Ana 300 LEYLNLKDN-SIHVSSHSFYGLSSVKHLNLINSLTG---------KIEDFSFHWLYHLEYLLMDNNNFPRITTNMFTGLNNLKYLSLSNCNINLQRITNT

Cai 300 LEYLNLKDN-IIHVSSRSFYGLSSIKHLNLINSLTG---------KIEDFSFHWLYHLEYLLMDNNNFPRITTNMFTGLNNLKYLSLSNCNINLQRITNT

Gal 246 LEYLNLEDNNIINVSSHLFYGLSSITHLNLINSLSG---------KIEDFSFQWLHHLEYLIMDNNNFPRITTNMFTGLKNLKYLSLYNCNTNLQRITNK

Hom 300 LEYFFLEYNNIQHLFSHSLHGLFNVRYLNLKRSFTKQSISLASLPKIDDFSFQWLKCLEHLNMEDNDIPGIKSNMFTGLINLKYLSLSNSFTSLRTLTNE

Mus 301 LRYLSLEYNNIQRLSPRSFYGLSNLRYLSLKRAFTKQSVSLASHPNIDDFSFQWLKYLEYLNMDDNNIPSTKSNTFTGLVSLKYLSLSKTFTSLQTLTNE

Pek 390 TFLSLANSSLQVLNLTKTRIYTIESGAFSCLGHLKILDLGLNEISQELTGHEFKGLNNIQYIYLSYNKNLTLQSESFSFVPSLRMLMLRKVGCSNLAISP

Ana 390 TFLSLANSSLQVLNLTKTRIYTIESGAFSCLGHLKILDLGLNEISQELTGHEFKGLNNIQYIYLSYNKNLTLRSESFSFVPSLRMLMLRKVGCSNLAISP

Cai 390 TFLSLANSSLQVLNLTKTRIYTIESGAFSCLGHLKILDLGLNEISQELTGHEFKGLSNIQYIYLSYNKNLTLRSESFSFVPSLRMLMLRKVGCSNLAISP

Gal 337 TFVSLANSSLQVLNLTKTRISTVESGAFSSLGQLKILDLGLNEINQELTGHEFEGLNNIEYIYLSYNKNVTLRSESFIFVPSLRKLMLRKVGCNNLAISP

Hom 400 TFVSLAHSPLHILNLTKNKISKIESDAFSWLGHLEVLDLGLNEIGQELTGQEWRGLENIFEIYLSYNKYLQLTRNSFALVPSLQRLMLRRVALKNVDSSP

Mus 401 TFVSLAHSPLLTLNLTKNHISKIANGTFSWLGQLRILDLGLNEIEQKLSGQEWRGLRNIFEIYLSYNKYLQLSTSSFALVPSLQRLMLRRVALKNVDISP

Pek 490 SPFHPLQNLTVLDISNNNIANIKEDLFDGLHKLDILNLQHNNLARLWKHANPGGPVLFLKDLPNLHVLNLKSNGFDEIPVQVFKGLFQLKYLDLGSNNLN

Ana 490 SPFHPLQNLTVLDISNNNIANIKEDLFDGLHKLDILNLQHNNLARLWKHANPGGPVLFLKDLPNLHVLNLKSNGFDEIPVQVFKGLFQLKYLDLGSNNLN

Cai 490 SPFHPLQNLTVLDISNNNIANIKEDLFDGLHKLDILNLQHNNLARLWKHANPGGPVLFLKDLPNLRVLNLKSNGFDEIPVQVFKGLFQLKYLDLGSNNLN

Gal 437 SPFHPLRNLTVLDISNNNIANIKEDLFNGLHELDILNLQHNNLARLWKCANPGGPVLFLKDVPNLHILNLKSNGFDEIPVHVFKGLHQLKDLDLGSNNLN

Hom 500 SPFQPLRNLTILDLSNNNIANINDDMLEGLEKLEILDLQHNNLARLWKHANPGGPIYFLKGLSHLHILNLESNGFDEIPVEVFKDLFELKIIDLGLNNLN

Mus 501 SPFRPLRNLTILDLSNNNIANINEDLLEGLENLEILDFQHNNLARLWKRANPGGPVNFLKGLSHLHILNLESNGLDEIPVGVFKNLFELKSINLGLNNLN

Pek 590 LLPATLFDDQTSLNSLNLQKNLITSVEEEVFGSAFKNLRKLEMDSNPFDCTCESIAWFASWLNATQAYIPGLQSQYICNTPPKYHGTLVLHFDTSVCKDS

Ana 590 LLPATLFDDQTSLNSLNLQKNLITSVEEEVFGSAFKNLRKLEMDSNPFDCTCESIAWFASWLNATQAYIPGLQSQYICNTPPKYHGTLVLHFDTSACKDS

Cai 590 LLPATLFDDQTSLNSLNLQKNLITSVEEEVFGSAFKNLRKLEMDSNPFDCTCESIAWFASWLNATQAYIPGLQSQYICNTPPKYHGIPVLHFDSSACKDS

Gal 537 LLPATLFDDQTSLNTLNLQKNLITSVEENVFGPAFKSLRTLEMDFNPFDCTCESIAWFASWLNDTQAYIPGLQSQYICNTPPKYHGTLVLHFDTSACKDS

Hom 600 TLPASVFNNQVSLKSLNLQKNLITSVEKKVFGPAFRNLTELDMRFNPFDCTCESIAWFVNWINETHTNIPELSSHYLCNTPPHYHGFPVRLFDTSSCKDS

Mus 601 KLEPFIFDDQTSLRSLNLQKNLITSVEKDVFGPPFQNLNSLDMRFNPFDCTCESISWFVNWINQTHTNISELSTHYLCNTPHHYYGFPLKLFDTSSCKDS

Pek 690 APFKLLFMITTTAVMLLIFVVLLIHFEGWRIAFYWNISVNRILGFKELDRLQEEYDYDAYIIHARHDRNWVLKNFISLEKNKQFEVRFCLEERDFEAGVS

Ana 690 APFKLLFMITTTAVMLLIFIVLLIHFEGWRIAFYWNISVNRILGFKELDRLQEEYDYDAYIIHARHDRNWVLKNFISLEKNKQFEVRFCLEERDFEAGVS

Cai 690 APFKLLFMITTTVVMLLIFIVLLIHFEGWRIAFYWNISVNRILGFKELDRLQEEYDYDAYIIHARHDRNWVLKNFISLEKNKQFEVRFCLEERDFEAGIS

Gal 637 APFKLLFLITTTAVMQFMFIVLLIHFEGWRIAFYWNISINRILGFKELDRLPGVFDYDAYVIHARKDTNWVLTNFTTLEENEQFQVKFCLEERDFEAGIS

Hom 700 APFELFFMINTSILLIFIFIVLLIHFEGWRISFYWNVSVHRVLGFKEIDRQTEQFEYAAYIIHAYKDKDWVWEHFSSMEKEDQ-SLKFCLEERDFEAGVF

Mus 701 APFELLFIISTSMLLVFILVVLLIHIEGWRISFYWNVSVHRILGFKEIDTQAEQFEYTAYIIHAHKDRDWVWEHFSPMEEQDQ-SLKFCLEERDFEAGVL

Pek 790 EFEAIINSIRRSRKIIFVVTEHLLKDPWCKKFKVYHAVQQAIEQSRDSIILVFLHDIQDYKLNHALCLRRGMFRSRCILKWPAQKERVNAFHQQLVMALK

Ana 790 EFEAIINSIRRSRKIIFVVTEHLLKDPWCKKFKVYHAVQQAIEQSRDSIILVFLHDIQDYKLNHALCLRRGMFRSRCILKWPAQKERVNAFHQQLVMALK

Cai 790 EFEAIINSIRRSRKIIFVVTEHLLKDPWCKKFKVYHAVQQAIEQSRDSIILVFLHDIQDYKLNHALCLRRGMFRSRCILKWPAQKERVNAFHQQLVMALK

Gal 737 EFEAIINCIRRSRKIIFIVTEHLLQDPWCRKFKVHHALQQAIEQSRDSIILIFLHNIQDYKLNHALCLRRGMFRSCCILNWPVQKERINAFHQQLMMALK

Hom 799 ELEAIVNSIKRSRKIIFVITHHLLKDPLCKRFKVHHAVQQAIEQNLDSIILVFLEEIPDYKLNHALCLRRGMFKSHCILNWPVQKERIGAFRHKLQVALG

Mus 800 GLEAIVNSIKRSRKIIFVITHHLLKDPLCRRFKVHHAVQQAIEQNLDSIILIFLQNIPDYKLNHALCLRRGMFKSHCILNWPVQKERINAFHHKLQVALG

Pek 890 SNSKAR

Ana 890 SNSKAR

Cai 890 SNSKAR

Gal 837 SNSK--

Hom 899 SKNSVH

Mus 900 SRNSAH

Signal Peptide LRR Transmembrrane Domain

TIR domain

**Figure 2**. Amino acid sequence alignment of duTLR3 with *Anas platyrhynchos*, *Cairina moschata*, *Gallus*, *Homo* *sapiens*, and *Mus* *musculus* TLR3 proteins. Alignment was performed using the CLUSTAW program and edited with BOXSHADE. Black boxes indicate amino acid identity; gray boxes indicates similarity (50% threshold). LRR, leucine rich repeat. TIR, toll-interleukin 1 receptor signaling domain. The TLR3 sequences are shown for Muscovy duck (Cai), human (Hom), mouse (Mus), chicken (Gal), Jinding duck (Ana), and peking duck (Pek)
